# Recent Advances in Developing Cell-Free Protein Synthesis Biosensors for Medical Diagnostics and Environmental Monitoring

**DOI:** 10.3390/bios15080499

**Published:** 2025-08-03

**Authors:** Tyler P. Green, Joseph P. Talley, Bradley C. Bundy

**Affiliations:** Department of Chemical Engineering, Brigham Young University, Provo, UT 84602, USA; tpg23@byu.edu (T.P.G.); talleyj2@byu.edu (J.P.T.)

**Keywords:** cell-free protein synthesis, biosensor, synthetic biology, environmental monitoring, medical diagnostics, point-of-care testing, paper-based detection, lyophilization, multiplexed sensing

## Abstract

Cell-free biosensors harness the selectivity of cellular machinery without living cells’ constraints, offering advantages in environmental monitoring, medical diagnostics, and biotechnological applications. This review examines recent advances in cell-free biosensor development, highlighting their ability to detect diverse analytes including heavy metals, organic pollutants, pathogens, and clinical biomarkers with high sensitivity and specificity. We analyze technological innovations in cell-free protein synthesis optimization, preservation strategies, and field deployment methods that have enhanced sensitivity, and practical applicability. The integration of synthetic biology approaches has enabled complex signal processing, multiplexed detection, and novel sensor designs including riboswitches, split reporter systems, and metabolic sensing modules. Emerging materials such as supported lipid bilayers, hydrogels, and artificial cells are expanding biosensor capabilities through microcompartmentalization and electronic integration. Despite significant progress, challenges remain in standardization, sample interference mitigation, and cost reduction. Future opportunities include smartphone integration, enhanced preservation methods, and hybrid sensing platforms. Cell-free biosensors hold particular promise for point-of-care diagnostics in resource-limited settings, environmental monitoring applications, and food safety testing, representing essential tools for addressing global challenges in healthcare, environmental protection, and biosecurity.

## 1. Introduction

Living cells serve as some of nature’s most versatile biochemical factories, capable of executing highly coordinated molecular processes essential for sustaining life. Cells are self-sustaining units that regulate gene expression, synthesize proteins, and respond to environmental stimuli, making them invaluable tools in biotechnological research. Scientific advancements have leveraged the natural capabilities of cells for applications ranging from pharmaceutical production to environmental sensing [1,2].

Cell-based biosensors integrate living cells with analytical systems to offer unique advantages over traditional sensing methods [3]. These biosensors have wide applications due to the natural selectivity of biological recognition elements like receptors, enzymes, and genetic circuits, enabling detection of analytes ranging from toxins to pathogens and metabolic biomarkers [4]. Cell-based biosensors have demonstrated significant promise in detecting disease markers for early diagnosis in medical applications [5], monitoring heavy metals and organic contaminants in environmental safety [6], and continuously assessing nutrient availability to optimize bioprocessing [7]. However, despite their versatility, cell-based biosensors face several critical limitations: stringent viability requirements, slow response times, cell-wall transport inhibition, and susceptibility to external stressors that may compromise functionality [8].

**Figure 1:**
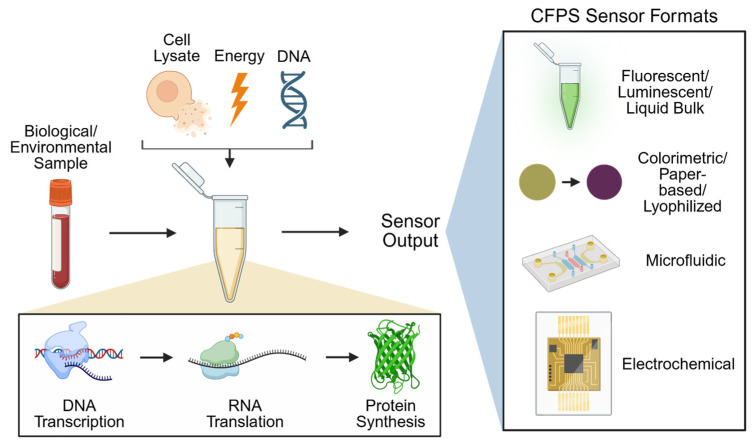
Design and applications of cell-free protein synthesis (CFPS) in biosensing. Sensor formats depicted include fluorescent [14], luminescent [15], liquid bulk [7], colorimetric [16], paper-based [17], lyophilized [18], microfluidic [19], and electrochemical [20].

Cell-free protein synthesis (CFPS) has emerged as a transformative technology that extracts and repurposes the essential biochemical machinery of cells to produce desired proteins without maintaining cell viability [9]. CFPS systems typically consist of purified ribosomes, transcription and translation factors, energy sources, and cofactors that enable protein production independent of cellular growth [10]. The historical evolution and biochemical utility of key additives used in cell-free systems have been extensively reviewed, providing insight into the development of optimized reaction mixtures [11]. By eliminating constraints associated with living cells, CFPS offers a highly tunable and efficient platform for biosensing applications across diverse fields (Figure 1). The potential of these systems has expanded dramatically in recent years, with applications extending from biosensing to biomanufacturing, as highlighted in recent comprehensive reviews [9,12,13].

In this article, we review recent advancements in the development of cell-free biosensors across three key application domains: environmental detection, medical diagnostics, and biotechnological tools. We analyze technological advances in CFPS optimization, including strategies for enhancing yield, sensitivity, and field deployment capabilities. We explore the integration of synthetic biology with cell-free systems to create advanced biosensors with complex signal detection and multiplexing capabilities. Finally, we examine emerging platforms and materials advancing the field and discuss future trends likely to shape cell-free biosensor development. Throughout, we highlight how these technologies overcome limitations of traditional cell-based approaches while offering versatility for detecting diverse analytes in various settings, from resource-limited environments to sophisticated laboratory applications.

## 2. Cell-Free Biosensors for Environmental and Medical Applications

### 2.1. Environmental Detection

Cell-free biosensors have demonstrated utility as tools for environmental monitoring due to their ability to detect various contaminants with high sensitivity and specificity while offering advantages over traditional detection methods. These biosensors leverage the flexibility of cell-free systems to operate in environments that would otherwise be toxic to living cells and can be designed for field deployment through preservation methods such as lyophilization and paper-based formats [5,21].

Heavy metal detection represents one of the earliest and most successful applications of cell-free biosensors. Gräwe et al. [22] developed a paper-based system for detecting mercury using a dual-filter approach with smartphone readout, achieving detection limits as low as 6 μg/L. Similarly, Jung et al. [6] created the ROSALIND platform capable of detecting copper, lead, and fluoride in water samples. Gupta et al. [14] designed a cell-free optical biosensor for mercury detection utilizing the merR gene incorporated into plasmid DNA constructs with reporter genes (firefly luciferase and emerald green fluorescent protein). Their biosensor achieved a detection limit of 1 ppb for mercury in water samples. This plasmid DNA-based approach demonstrated benefits when dealing with higher mercury concentrations that prove toxic to whole-cell biosensors, with additional refinements like pH adjustment and chelating agents further improving sensitivity. Early pioneering work by Zhang and Ruder [23] also demonstrated the potential of cell-free synthetic biological systems for environmental biosensing, highlighting the use of biotin detection in cell–cell-free systems to serve as environmental reporters.

More recently, Zhang et al. [24] developed a cell-free paper-based biosensor dependent on allosteric transcription factors (aTFs) for the on-site detection of harmful metals Hg^2+^ and Pb^2+^ in water. This approach achieved impressive detection limits of 0.5 nM for Hg^2+^ and 0.1 nM for Pb^2+^, with recoveries ranging from 91% to 123% for actual water samples. Advancements in biosensor engineering have significantly improved detection capabilities. Ekas et al. [25] developed a cell-free platform for engineering aTF biosensors with improved sensitivity, selectivity, and dynamic range. By applying this platform to engineer PbrR mutants, they achieved a shift in the limit of detection from 10 μM to 50 nM lead, demonstrating the potential for detecting lead at the legal limit. Beabout et al. [26] demonstrated systematic optimization approaches for heavy metal sensors, achieving detection limits below World Health Organization recommended maximum concentrations for arsenic (10 μg/L) and mercury (6 μg/L) through careful tuning of transcription factor concentrations and cell-free system selection. A notable application in this area involves monitoring arsenic using genetically encoded biosensors, as demonstrated by Wang et al. [27], who explored the role of evolved regulatory genes in improving sensing capabilities.

Beyond metals, cell-free systems have been adapted for detecting organic pollutants including pesticides and antibiotics. Silverman et al. [28] designed a cell-free atrazine biosensor by combining a cyanuric acid biosensor with a reconstituted metabolic pathway. This work built on an earlier design of a transcriptional biosensor for portable, on-demand detection of cyanuric acid [29]. For antibiotic detection, Dong et al. [30] created a riboswitch-based cell-free biosensor for the broad-spectrum detection of tetracyclines. This system used artificially screened tetracycline RNA aptamers to control the expression of reporter genes, achieving detection limits of 0.47, 0.079, 0.084, and 0.43 μM for tetracycline, oxytetracycline, chlortetracycline, and doxycycline, respectively. The system allowed for qualitative detection of tetracyclines in milk samples at concentrations as low as 1 μM. Earlier work by Duyen et al. [31] demonstrated paper-based colorimetric biosensors for detecting antibiotics that inhibit bacterial protein synthesis, providing a foundation for these advancements.

A significant breakthrough in biosensor technology addresses the detection of biological warfare agents. Park et al. [32] developed an innovative cell-free biosensor that transforms the 16S rRNA of targeted pathogens into detectable protein molecules. By integrating retroreflective Janus particles for signal amplification, the system achieved exceptional sensitivity, detecting 16S rRNA at femtomolar levels. The method demonstrated remarkable specificity for multiple dangerous pathogens, including *B. anthracis*, *F. tularensis*, *Y. pestis*, *B. Pseudomallei*, and *B. abortus.* Their approach utilized sensor DNAs engineered to produce fluorescent proteins with distinct spectral characteristics, enabling the simultaneous identification of multiple pathogenic species in a single assay.

The integration of metabolic pathways with biosensing circuits has dramatically expanded the range of detectable compounds. Voyvodic et al. [33] pioneered a plug-and-play approach combining synthetic metabolic cascades with transcription factor-based networks, enabling detection of molecules with no corresponding natural transcription factors. This work was complemented by Koch et al. [34], who reviewed the development of custom-made transcriptional biosensors specifically for metabolic engineering applications. Efforts to reduce costs and improve accessibility have been critical for practical adoption, with Arce et al. [35] developing low-cost cell extracts that reduced expense by two orders of magnitude while maintaining comparable performance to commercial systems.

The development of portable biosensors represents a significant advancement for field applications. Lin et al. [36] demonstrated portable environment-signal detection biosensors with cell-free synthetic biosystems, highlighting their potential for on-site monitoring in challenging environments. These systems offer unique advantages for detecting environmental contaminants in resource-limited settings.

Table 1 summarizes the performance characteristics of representative CFPS biosensors developed for environmental monitoring applications, highlighting detection capabilities for heavy metals, organic pollutants, and biological contaminants.

### 2.2. Medical Diagnostics

Cell-free biosensors have gained significant attention in medical diagnostics due to their potential for rapid, sensitive, and specific detection of biomarkers and pathogens in clinical samples. These biosensors enable point-of-care testing with minimal equipment requirements, making them particularly valuable for resource-limited settings.

The development of hormone-responsive biosensors represents a significant advancement in the field. Salehi et al. [37] pioneered a cell-free protein synthesis approach for detecting thyroid receptor β-specific endocrine disruptors using an engineered allosterically activated fusion protein. Building on this work, Salehi et al. [38] developed the Rapid Adaptable Portable In vitro Detection (RAPID) platform for detecting estrogenic compounds in human blood and urine samples with detection times significantly faster than traditional cellular assays. Hunt et al. [39] further elucidated the mechanistic underpinnings of these hormone biosensors, demonstrating that intein-mediated catalytic cleaving is critical for hormone-dependent signal generation. Through mathematical simulation and optimization, they achieved a three-fold improvement in assay readable window and twofold improvement in signal strength. Subsequently, Hunt et al. [40] reduced costs by 60% by replacing the β-lactamase/nitrocefin reporter system with various β-galactosidase configurations while maintaining effective detection of thyroid receptor ligands.

The direct detection of clinical biomarkers from patient samples represents a crucial capability for diagnostic applications. Wen et al. [41] demonstrated this by developing a cell-free biosensor for detecting quorum sensing molecules in *P. aeruginosa*-infected respiratory samples from cystic fibrosis patients. Their system quantitatively measured 3-oxo-C12-HSL at nanomolar levels in sputum samples, with results comparable to liquid chromatography–mass spectrometry (LC-MS) measurements. Hunt et al. [7] extended this clinical utility by creating a system for detecting glutamine in human serum. By inhibiting glutamine synthetase with methionine sulfoximine, they developed a sensor with a detection range well suited to physiological glutamine concentrations.

Recent innovations have focused on expanding the range of detectable clinical analytes. Beabout et al. [42] engineered a transcription factor-based biosensor for bile acids in complex matrices including fecal water, wastewater, and serum, achieving approximately three orders of magnitude increase in deoxycholic acid sensitivity compared to cellular systems. Piorino et al. [16] developed a cell-free biosensor for assessment of hyperhomocysteinemia that detects physiologically relevant concentrations in plasma via colorimetric output in under 1.5 h. Free et al. [18] addressed signal resolution challenges by engineering a paper-based cell-free glutamine biosensor through genetic engineering, metabolic engineering, and process optimization. Future work by Talley et al. [43] further expanded the utility of this sensor by exploring a novel aspartate-based sensor for glutamine, eliminating background signal detection. Free et al. [17] additionally developed at-home dilution and filtration methods for paper-based colorimetric biosensing in human blood.

The integration of CRISPR technology with cell-free systems represents a cutting-edge direction in biosensor development. Wang et al. [20] developed an electrochemical assay combining aTFs and CRISPR-Cas14a for detecting progesterone. By optimizing probe configuration, they achieved detection in the range of 67 pM to 0.33 μM, requiring only 2 μL of sample without complex pretreatment. Hong et al. [44] demonstrated complementary approaches using ultraspecific riboregulators for precise and programmable detection of mutations, achieving single-nucleotide discrimination capabilities that enhance the specificity of nucleic acid-based biosensors. Chen et al. [45] developed a cell-free fluorescence biosensor for pentachlorophenol using the aTF NalC. By combining in vitro transcription with Nucleic Acid Sequence-Based Amplification, they achieved a limit of detection of 0.002 μM with excellent specificity and recovery rates between 101% and 114% in environmental samples.

Enabling point-of-care diagnostics often requires overcoming challenges associated with processing complex biological samples. McSweeney et al. [46] addressed this by developing a modular cell-free protein biosensor platform using split T7 RNA polymerase for protein detection. Their T7 RNA polymerase-linked immunosensing assay enables detection of various protein biomarkers in serum and saliva with a colorimetric readout within one hour. Narahari et al. contributed to this field by developing portable sample processing methods specifically for molecular assays in Zika virus diagnostics.

Novel clinical applications continue to emerge, as demonstrated by Yenilmez et al. [47], who developed a new biosensor for fetal RHD detection from circulating cell-free fetal DNA in maternal plasma, highlighting the potential of this technology for non-invasive prenatal diagnostics. Regarding Zika virus detection, Narahari et al. [48] developed portable sample processing methods for molecular assays in Zika virus diagnostics and Chen et al. [49] presented a cell-free toehold switch biosensor which detects Zika virus with high specificity and sensitivity. The future of artificial biosensor technology in biomedical applications looks promising, with Gaba et al. [50] projecting significant advances in diagnostic capabilities and integration with other technologies to address emerging healthcare challenges.

Table 2 summarizes representative CFPS biosensors for medical diagnostic applications, emphasizing systems that have demonstrated clinical validation, detection in physiological samples, or potential for point-of-care implementation.

## 3. Technological Advances and Optimization Strategies in Cell-Free Protein Synthesis for Biosensor Development

### 3.1. Optimization of Yield and Sensitivity

CFPS systems offer distinct advantages for biosensor development by enabling direct access to reaction components without transmembrane transport limitations and allowing precise introduction of potentially toxic analytes [12]. Enhancing yield and sensitivity through systematic optimization has significantly advanced CFPS biosensor performance across multiple fronts.

Reaction condition optimization has proven critical for maximizing CFPS performance. Systematic approaches to extract preparation have been critical for reproducible performance. Cole et al. [51] provided comprehensive methodologies for prokaryotic extract preparation, while Dopp and Reuel [52,53] developed scalable extraction protocols and identified methods to reduce experimental variability, including optimized extract preparation and careful reaction mixing procedures. Banks et al. [54] identified critical interactions between 20 components using a statistical Design of Experiments approach, finding that magnesium glutamate, 3-phosphoglyceric acid, polyethylene glycol, and cell extract concentration most significantly influenced protein synthesis, achieving a 400% improvement in yield with enhanced cross-batch robustness. Spice et al. [55] applied a minimized DOE strategy to *P. pastoris* cell-free systems, identifying HEPES buffer, potassium glutamate, and creatine phosphate as critical components, resulting in 4.8-fold increases in firefly luciferase production without requiring sophisticated equipment. This optimization approach has shown significant promise for engineering high-yield cell-free protein synthesis platforms, as demonstrated by Aw and Polizzi [56], who used biosensor-assisted approaches to enhance system performance and overcome a bottleneck in ribosome content.

The physical–chemical environment substantially impacts reaction kinetics. Vezeau and Salis [57] demonstrated that common cosolutes like PEG-8000 and Ficoll-400 differentially affect transcription and translation mechanisms, with PEG-8000 and Ficoll-400 increasing translation initiation rates (about 8-fold and 3-fold, respectively) while all tested cosolutes negatively impacted translation elongation, revealing important trade-offs for optimizing specific biosensor applications. Chushak et al. [58] characterized riboswitch activation kinetics, showing that kinetic trapping mechanisms rather than thermodynamic equilibrium govern activation, with only a portion of mRNA molecules reaching the “ON” state even at high ligand concentrations.

Genetic element optimization has further enhanced biosensor performance. Copeland et al. [59] discovered that computational tools for predicting translation rates often fail in cell-free environments, with mRNA secondary structure significantly impacting ribosome binding site accessibility. They found that codon optimization increased reporter protein expression 1.3-fold for mNeonGreen, while the g10 leader sequence from T7 phage outperformed computationally designed ribosome binding sites. In the same study, they characterized mNeonGreen as an alternative to superfolder GFP, demonstrating 2.6 times higher fluorescence output and a 2.1-fold higher RFU/μM ratio despite slower maturation kinetics, ultimately achieving a 2.32-fold greater fluorescence fold change after complete maturation. These findings emphasize the importance of empirical validation in CFPS environments.

Alternative signal output strategies have accelerated detection capabilities. Li et al. [60] coupled ribozyme cleavage reactions with genetic circuits, using ribozymes as the transcription output to catalytically cleave fluorescent substrate probes. This approach eliminated time-consuming translation steps required by fluorescent protein reporters while achieving signal amplification through the catalytic nature of the ribozyme, enabling rapid and sensitive detection of small molecules. Novel approaches like suppressor tRNA-based biosensors have also been developed, as described by Ogawa [61], providing alternative strategies for detecting various analytes with high specificity.

CFPS systems have been adapted for complex biological samples through innovative approaches. Soltani et al. [62] produced murine RNase Inhibitor (m-RI) in E. coli lysate-based CFPS, essential for inhibiting RNases in human fluids that would otherwise degrade RNA components. By optimizing reaction parameters and adding GroEL/ES folding chaperones, they achieved soluble m-RI titers approaching 750 μg/mL in 2 h, reducing reagent costs by approximately 90% compared to commercial inhibitors. Soltani and Bundy [63] further streamlined this process by overexpressing m-RI during extract preparation, creating CFPS systems capable of functioning in saliva, serum, and urine at substantially reduced costs.

Smith et al. [64] simplified CFPS preparation by combining DNA template and cell extract production into a single fermentation process. Their “just add small molecules” method eliminated separately purified DNA by using cells harboring target plasmids to create cell extracts, reducing reagent costs by more than half while maintaining sufficient intact plasmid for transcription.

Advanced applications of CFPS include high-yield production systems with potential therapeutic applications. Hunt et al. [65] demonstrated the engineering of CFPS for high-yield production and human serum activity assessment of asparaginase, advancing the potential for on-demand treatment of acute lymphoblastic leukemia. This work illustrates how optimized cell-free systems can be used not only for sensing but also for therapeutic protein production.

### 3.2. Preservation and Field Deployment Strategies

The practical application of cell-free biosensors outside laboratory settings requires effective preservation methods and field deployment strategies. These technologies have advanced significantly, enabling the use of sophisticated molecular detection systems in resource-limited environments and field conditions. Lyophilization (freeze-drying) has emerged as a fundamental technique for creating shelf-stable CFPS reactions. Hunt et al. [21] demonstrated that lyophilized cell-free expression systems maintain functionality and can be distributed without cold-chain requirements, enabling “just-add-water” functionality for on-demand protein synthesis. This approach eliminates the need for specialized equipment and trained personnel, making it particularly valuable for remote settings. The preserved reactions can be rapidly reactivated with minimal preparation, producing measurable results in as little as one hour.

**Figure 2:**
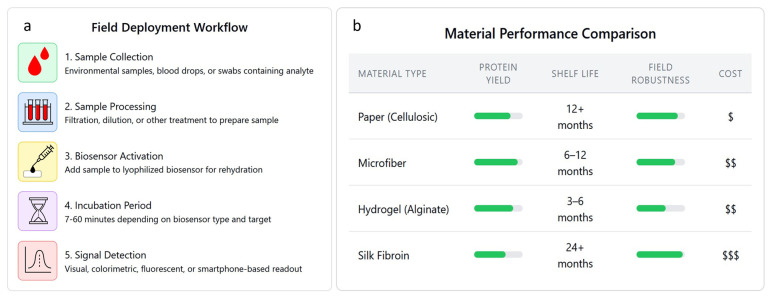
Field deployment strategies for cell-free protein synthesis (CFPS) biosensors (**a**) field deployment workflow from sample collection to signal detection, (**b**) comparison of materials used for CFPS biosensor integration and preservation.

The workflow for field deployment of preserved cell-free biosensors and a brief comparison of materials which commonly constitute them are described in Figure 2. Preserved cell-free biosensors are deployed by collecting and processing a sample, activating the biosensor, and then measuring signal detection after an incubation period (Figure 2a).

Material selection and rehydration protocols significantly impact performance, shelf life, robustness, and cost of lyophilized cell-free systems (Figure 2b). Blum et al. [66] evaluated 32 paper-like materials and five hydrogel matrices, demonstrating that material selection significantly impacts system performance after lyophilization. They found that synthetic polymer-based materials were largely incompatible with cell-free reactions, while cellulosic and microfiber materials performed well, particularly when lyophilized. The superior performance of cellulosic materials can be attributed to their hydrophilic nature and porous structure [67], which facilitates uniform water removal during lyophilization while providing a stable matrix that prevents protein denaturation and maintains the integrity of nucleic acid components. Microfiber materials offer similar advantages through their high surface area-to-volume ratio [68], which promotes rapid water sublimation and creates an optimal microenvironment that preserves enzyme activity during storage. Modulating rehydration volume of lyophilized reactions yielded reaction speed increases up to twofold, with optimal rehydration ratios identified for both lysate (2:1) and purified (1.5:1) systems. The mechanism underlying improved rehydration performance involves achieving optimal molecular crowding conditions that enhance protein–protein interactions and enzymatic kinetics [69], while preventing dilution effects that could compromise translation efficiency.

Paper-based platforms have emerged as effective carriers for field-deployable biosensors. Hunt et al. [15] developed a paper-based cell-free toehold switch biosensor for detecting SARS-CoV-2 RNA in human saliva. Their system used lyophilized CFPS and toehold switch riboregulators on paper supports activated by saliva addition, generating visible signals in as little as seven minutes with estimated costs below USD 0.50 per test. Additionally, Carr et al. [70] advanced the field by interfacing gel switch resonators with cell-free toehold switches for mail-in SARS-CoV-2 detection, addressing distribution and usability challenges.

Sample processing innovations have addressed key challenges in field applications. Free et al. [17] developed user-friendly dilution and filtration methods for blood collection using inexpensive, readily available materials for a colorimetric glutamine biosensor that processed 50 μL blood drop samples in under 30 min. They found that BSA-treated paper towels could serve as low-cost alternatives to expensive filtration membranes for passive serum extraction. The effectiveness of BSA-treated paper towels stems from the protein’s ability to block non-specific binding sites and create a selective permeability barrier that allows small molecule analytes to pass while retaining larger interfering components [71]. Free et al. [18] further enhanced this platform through genetic engineering, metabolic engineering, and process optimization to overcome background limitations, extending the detection window from 15 to over 60 min and allowing clearer distinction of intermediate responses.

Integration with microfluidic and smartphone platforms has enhanced usability. Tonooka [19] integrated lyophilized CFPS systems into microfluidic devices that require only 1 µL sample volumes, providing operational simplicity. The miniaturization benefits arise from reduced diffusion distances and enhanced mass transfer rates in microscale environments, which accelerate reaction kinetics while minimizing reagent consumption [72]. Nelis et al. [73] explored smartphone-based sensing devices (SBDs) for in situ analysis, highlighting how these overcome key challenges including miniaturization, power consumption, and user-friendly operation.

While these engineering innovations improved portability and accessibility, a parallel challenge has been expanding the range of detectable analytes beyond nucleic acids. McSweeney et al. [46] introduced T7 RNA polymerase-linked immunosensing assay (TLISA), combining split T7 RNA polymerase with protein-binding affinity domains for versatile protein detection. By fusing different affinity domains to split T7 RNA polymerase fragments, they demonstrated detection of various protein targets with minimal protocol optimization, producing colorimetric readouts within 1 h in serum and saliva while maintaining functionality after lyophilization.

While lyophilization remains the most common preservation method, alternative strategies offer complementary approaches with unique advantages. Silk fibroin encapsulation achieves exceptional stability through its unique protein structure that forms a protective glass-like matrix around biosensor components [74], providing superior mechanical protection and moisture barrier properties compared to conventional lyophilization. Drachuk et al. demonstrate that silk fibroin encapsulation, as previously mentioned, achieves exceptional stability without requiring cold-chain maintenance [75]. This approach has particular relevance for applications requiring extended shelf life in resource-limited settings where refrigeration may be unavailable.

## 4. Integration of Synthetic Biology with Cell-Free Systems for Advanced Biosensor Applications

### 4.1. Complex Signal Processing and Multiplexed Systems

As cell-free systems continue to evolve, they are increasingly positioned as accessible platforms that span the range from biosensing to biomanufacturing [12]. The integration of synthetic biology with cell-free protein synthesis (CFPS) has enabled increasingly sophisticated biosensors capable of complex signal detection and multiplexed sensing. These advanced systems incorporate genetic circuits, engineered molecular components, and novel detection mechanisms to simultaneously detect multiple analytes or process complex environmental signals.

Signal amplification represents a significant advancement in CFPS biosensor technology, addressing the critical need for improved sensitivity and reduced response times. The previously discussed ribozyme-based amplification strategy by Li et al. [60] demonstrated how catalytic RNA elements can be integrated into more complex detection platforms, illustrating the broader trend toward leveraging natural biological amplification mechanisms to overcome sensitivity limitations in cell-free systems. Building on this concept, Li et al. [76] developed a cell-free biosensor signal amplification circuit utilizing polymerase strand recycling, leveraging T7 RNA polymerase off-target transcription to recycle nucleic acid inputs within DNA strand displacement circuits. This innovation improved detection limits by 10-fold, reaching submicromolar levels for tetracycline (0.025 µM) and zinc (0.1 µM).

Advanced multiplexed detection capabilities have been enhanced through electrochemical interfaces, as demonstrated by Mousavi et al. [77], who developed gene-circuit-based sensors with electrochemical readouts. Ma et al. [78] further advanced the field by engineering multi-arm RNA junctions that enable molecular logic operations unconstrained by input sequence, expanding diagnostic versatility. Mathur et al. [79] developed hybrid nucleic acid–quantum dot assemblies as multiplexed reporter platforms for CFPS biosensors. Their system utilized differentially emissive quantum dot donors paired with dye-acceptors on DNA strands encoding specific enzyme cleavage sites, enabling simultaneous monitoring of multiple enzymatic activities using a common excitation wavelength but distinct emission spectra.

CRISPR-based detection systems have enhanced biosensor performance in challenging samples by enabling sophisticated signal processing. The previously mentioned work of Wang et al. [20] exemplifies how CRISPR-Cas systems can be integrated with other sensing elements to overcome common limitations. Their progesterone detection system employed longer sgRNA designs to overcome sequence folding issues that typically hamper detection efficiency, while the Cas14a protein’s collateral cleavage activity provided signal amplification. This innovative approach enabled rapid detection (within 1.5 h) even in complex biological matrices, demonstrating how advanced CRISPR-based signal processing can address practical challenges in clinical diagnostics.

Targeted detection of pathogens has advanced through innovative approaches. Park et al. [32] introduced a cell-free biosensor for the multiplexed detection of biological warfare agents that targets the 16S rRNA of pathogens and converts this detection into functional protein molecules. The modular design allows simultaneous identification of multiple pathogenic 16S rRNAs through customized reporter proteins, integrating with retroreflective Janus particles to achieve femtomolar sensitivity corresponding to tens of colony-forming units per milliliter of pathogenic bacteria.

The chemical detection range of cell-free biosensors has been substantially expanded through metabolic transducers. Voyvodic et al. [33] developed plug-and-play metabolic transducers that combine synthetic metabolic cascades with transcription factor-based networks to convert target molecules into detectable outputs. This system converted hippuric acid and cocaine into benzoic acid, which could be detected by the transcription factor BenR. These hybrid cell-free biosensors demonstrated fast response times, strong signal responses, and high dynamic range, functioning effectively in complex media including commercial beverages and human urine.

Membrane integration has further advanced biosensor capabilities. Peruzzi et al. [80] further explored transmembrane signal transduction in synthetic membranes using bacterial nitrate-sensing two-component systems. By altering the biophysical properties of the membrane containing the histidine kinase, they showed that performance and sensitivity could be tuned. Through protein engineering, they modified the sensing domain to generate sensors capable of detecting various ligands, demonstrating how membrane-augmented cell-free systems can characterize membrane–receptor interactions and engineer biosensors for environmental applications.

Table 3 presents CFPS biosensors that incorporate advanced signal processing capabilities, multiplexed detection systems, and signal amplification strategies to enhance sensitivity and enable simultaneous multi-analyte detection.

### 4.2. Novel Sensor Design Strategies

Cell-free protein synthesis provides a versatile platform for implementing novel sensor designs that expand detection capabilities beyond what is possible with traditional approaches. These innovative designs leverage the flexibility of cell-free systems to incorporate diverse recognition elements, signal processing mechanisms, and output strategies. The foundational work by Green et al. [81] in developing toehold switches as de novo-designed regulators of gene expression established a versatile platform for programmable biosensor design that has enabled many subsequent innovations.

The integration of mechanosensitivity with biosensing represents an innovative direction in the field. Majumder et al. [82] constructed mechanosensitive liposomes with biosensing capability, expressing the E. coli channel MscL and a calcium biosensor within synthetic liposomes. This system could sense both osmotic pressure through MscL activation and external calcium concentration through the fluorescent biosensor while exhibiting selective permeability based on molecular size. Garamella et al. [83] created an adaptive synthetic cell equipped with an inducible genetic circuit responsive to osmotic pressure changes through MscL. Their system combined mechanosensitivity, biosensing, and gene expression, where liposomes loaded with cell-free transcription–translation machinery could be induced with Isopropyl ß-D-1-thiogalactopyranoside (IPTG) when exposed to hypo-osmotic conditions, resulting in the expression of the bacterial cytoskeletal protein MreB.

Riboswitches have emerged as versatile platforms for CFPS biosensor design. Harbaugh et al. [84] engineered synthetic dopamine-responsive riboswitches for in vitro biosensing by converting dopamine-binding aptamers into functional riboswitches, achieving sensitive detection in human urine at physiologically relevant concentrations. Thavarajah et al. [85] demonstrated naturally occurring riboswitches for environmental sensing, developing a fluoride-responsive biosensor using the *B. cereus* crcB riboswitch capable of detecting fluoride above 2 ppm in both laboratory and field conditions. Dong et al. [30] applied this approach to create a broad-spectrum detection system for tetracyclines, with their paper-based and tube-based formats remaining stable at room temperature.

Split reporter systems have advanced CFPS biosensor capabilities. Kim et al. [86] introduced a split T7 switch-mediated system for target nucleic acids, applying the split T7 promoter to a three-way junction structure that selectively initiated transcription–translation only in the presence of targets, offering thousand-fold sensitivity improvements over toehold switch approaches with detection limits as low as 10 pM. Copeland et al. [87] employed a split mNeonGreen system, expressing only the small 11th β-strand (2.2 kDa) rather than the complete protein (25.7 kDa), increasing reporter unit production before CFPS reaction exhaustion. By splitting these proteins into two segments (1–10 and the 11th β-strand), they created systems capable of producing more reporter units before cell-free reaction cessation, significantly enhancing detection limits.

Metabolic sensing approaches have expanded detectable analyte ranges. Lee et al. [88] developed quantitative analysis of γ-aminobutyric acid (GABA) by combining enzymatic conversion with amino-acid-dependent CFPS, using GABA transaminase to convert GABA to alanine for incorporation into signal-generating proteins during synthesis. Silverman et al. [28] pioneered “metabolic biosensing” for atrazine detection by combining a cyanuric acid biosensor with a reconstituted atrazine-to-cyanuric acid metabolic pathway composed of protein-enriched bacterial extracts, detecting atrazine within an hour by manipulating ratios of enriched extracts.

Colorimetric reporters enhance field utility. Sharpes et al. [89] systematically evaluated seven enzymes and fifteen substrates for PURE cell-free system compatibility, identifying four enzymes (LacZ, GusA, CelB, and AES) and eight substrates that functioned effectively. They demonstrated that combining LacZ and GusA produced a third distinct color when both were present, enabling multiplexed sensors with unique visual outputs requiring no sophisticated instrumentation.

Table 4 highlights novel CFPS biosensor design strategies that leverage innovative recognition elements, split systems, and unconventional detection mechanisms to expand sensing capabilities.

## 5. Emerging Materials and Future Directions

### 5.1. Advanced Materials for Cell-Free Biosensors

Recent advances have significantly expanded the capabilities of cell-free biosensors by providing improved platforms for protein expression, analyte detection, and signal transduction. These innovations have addressed key limitations in traditional approaches while creating new opportunities for biosensor development.

Supported lipid bilayers (SLBs) have emerged as foundational platforms that maintain in vivo-like fluidity while facilitating integration with sensing modalities. Jayaram et al. [90] reviewed progress in this area, noting that polymer-cushioned SLBs have successfully incorporated proteins with improved mobility, though challenges remain in controlling cushion morphology and density. Native cell membrane-derived SLBs represent a significant advancement, capturing lipid, protein, and glycan species from live cells while maintaining transmembrane protein mobility and orientation, providing more accurate representations of cellular interactions without whole-cell complexity.

The integration of transmembrane proteins with organic electronic materials has demonstrated promising results for creating bioelectronic interfaces. Manzer et al. [91] developed a one-step integration of ion channels (MscL) into SLBs assembled on poly(3,4-ethylenedioxythiophene) polystyrenesulfonate (PEDOT:PSS), creating a dual-modality platform allowing both optical and electrical recording of small molecule transport. This approach confirmed that MscL channels adopt correct orientation, remain mobile in the SLB, and maintain activity on polyelectrolyte surfaces, enabling rapid assembly of bioelectronic platforms for diverse sensing applications.

Hydrogels provide another promising class of materials for CFPS biosensors. Benítez-Mateos et al. [92] demonstrated microcompartmentalization of CFPS in various hydrogel materials (alginate, collagen, laponite, and agarose) within μ-channel devices, providing advantages over spherical compartments including uniformity, ease of monitoring, and microfluidic integration. Their study showed that alginate hydrogels successfully produced proteins with yields of approximately 3.8 μg/mL, with the alginate enhancing CFPS performance through a crowding effect. Different hydrogel materials exhibited varying performance characteristics, with alginate and laponite showing excellent protein expression yields while collagen demonstrated lower efficiency.

Artificial cells incorporating lipid membranes have emerged as sophisticated biosensing platforms. Boyd et al. [93] demonstrated cell-free riboswitches encapsulated in lipid vesicles for detecting environmentally relevant fluoride concentrations. This approach combines cell-free detection with protective lipid membrane barriers that shield sensing components from degradative factors in complex samples. Membrane composition tuning enabled optimization of sensor sensitivity and dynamic range, while the encapsulation protected against external enzyme degradation, enabling detection in real-world water samples.

### 5.2. Future Challenges and Opportunities

Despite significant advances, several technological and practical limitations continue to challenge the widespread adoption of CFPS-based biosensors, particularly for clinical translation and field reliability. Standardization remains a critical barrier, as interlaboratory variability in extract preparation and reaction conditions can lead to inconsistent performance across different settings. Sample matrix interference poses another significant challenge, with complex biological fluids and environmental samples often containing components that inhibit protein synthesis or interfere with detection mechanisms. The limited shelf life of many CFPS components, even with lyophilization, constrains long-term storage and distribution in resource-limited settings where cold-chain infrastructure may be unreliable. Sensitivity limitations persist for certain analytes, particularly when competing with established analytical methods like LC-MS or ELISA that offer superior detection limits. Regulatory approval pathways for CFPS-based diagnostic devices remain unclear in many jurisdictions, creating uncertainty for clinical translation. Additionally, the complexity of optimizing reactions for diverse sample types and the need for specialized knowledge in synthetic biology may limit adoption by end-users in field settings. Cost considerations, while improved through simplified preparation methods, still present barriers for large-scale deployment in low-resource environments where these technologies could have the greatest impact.

**Figure 3:**
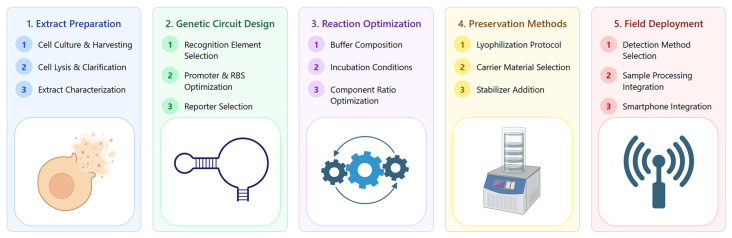
Comprehensive workflow for developing cell-free protein synthesis (CFPS) biosensors, showing the five key stages of development: extract preparation, genetic circuit design, reaction optimization, preservation methods, and field deployment.

CFPS biosensor development involves many interdependent workflow components (Figure 3) which have each advanced significantly as detailed in this review. However, continued innovation across these areas remains essential for successful commercial deployment.

Future developments in extract preparation may focus on standardizing protocols to reduce interlaboratory variability, as highlighted by Cole et al. [94], while simultaneously reducing costs and improving accessibility. The trend toward simplified preparation methods, exemplified by Smith et al. [64] with their “just add small molecules” approach, represents a promising direction for democratizing cell-free biosensor technology. Advanced extract engineering approaches, such as the overexpression of critical components like RNase inhibitors during extract preparation [63], will likely expand to incorporate other specialized proteins that enhance biosensor performance in challenging sample matrices. Future extract preparation strategies may also leverage machine learning approaches to optimize component ratios and identify novel additives that improve yield, stability, and reproducibility across different laboratory settings.

The next generation of genetic circuits will incorporate increasingly sophisticated signal processing capabilities, building on advances in riboswitch engineering [84], split reporter systems [86,87], and CRISPR-based detection platforms [20]. Future circuit designs will likely integrate multiple recognition elements to enable simultaneous detection of related analytes or provide built-in controls for sample quality assessment. The development of standardized genetic parts and modular circuit architectures will facilitate rapid prototyping and deployment of new biosensors for emerging targets. Additionally, the integration of machine learning approaches for circuit design optimization will accelerate the development of high-performance biosensors with predictable characteristics and reduced development timelines. Future innovation may benefit from insights gained in transcription factor-based biosensor development across diverse bacterial platforms, as reviewed by Kim et al. [95], to expand cell-free biosensor capabilities beyond current E. coli-dominated systems.

Systematic optimization approaches, such as those demonstrated by Banks et al. [54] and Spice et al. [55], could evolve to incorporate real-time monitoring and adaptive control systems that automatically adjust reaction conditions based on sample characteristics. The development of universal reaction formulations that perform consistently across diverse sample types and environmental conditions remains a critical challenge that will require continued research into cosolute effects [57] and kinetic mechanisms [58]. Advanced modeling approaches will facilitate predictive optimization, reducing the empirical testing required for new biosensor applications.

While lyophilization has emerged as the dominant preservation strategy [21], future developments may focus on alternative preservation methods that offer complementary advantages for specific applications. The silk fibroin encapsulation approach demonstrated by Drachuk et al. [75] represents one promising direction that may enable even longer shelf life and improved stability under extreme environmental conditions. The development of preservation methods that maintain biosensor activity while enabling rapid reactivation with minimal user intervention will be critical for point-of-care applications in resource-limited settings.

The future of field deployment emphasizes user experience design and integration with ubiquitous technologies. Building on smartphone integration demonstrated by Gräwe et al. [22] and microfluidic approaches by Tonooka [19], future deployments will likely incorporate augmented reality interfaces, automated result interpretation, and cloud-based data management systems. Digital integration presents both significant opportunities and technical challenges for CFPS biosensor deployment. Signal processing algorithms must be optimized for mobile platforms to handle diverse optical and electrochemical readouts while compensating for variations in ambient lighting, camera quality, and user technique. Machine learning approaches could enable automated image analysis and result interpretation, reducing user error and providing standardized quantitative outputs from colorimetric or fluorescent signals. Cloud-based platforms offer the potential for real-time data aggregation, epidemiological tracking, and remote expert consultation, but require robust cybersecurity protocols and standardized data formats to ensure patient privacy and regulatory compliance. User interface design becomes critical for non-expert users, necessitating intuitive workflows that guide sample collection, processing, and result interpretation while providing clear quality control indicators and error detection. Data sharing capabilities could enable population-level health monitoring and environmental surveillance networks, but must address challenges related to data ownership, privacy regulations, and interoperability between different biosensor platforms and healthcare systems. Sample processing innovations, such as those developed by Free et al. [17], may expand to address diverse sample types while maintaining simplicity for non-expert users. The integration of cell-free biosensors with Internet of Things (IoT) networks could enable continuous environmental monitoring and real-time alert systems, with edge computing capabilities reducing latency and enabling autonomous decision-making in remote locations. Future field deployment strategies will likely also address regulatory compliance and quality assurance challenges, incorporating built-in controls and standardized protocols that meet clinical and environmental monitoring requirements across different jurisdictions while ensuring seamless integration with existing healthcare IT infrastructure and environmental monitoring systems.

## 6. Conclusions

Cell-free biosensors represent a significant advancement in analytical technology, utilizing cellular machinery without the limitations of maintaining cell viability. This review has tracked the development of these systems from experimental prototypes to validated analytical tools with practical applications in environmental monitoring, clinical diagnostics, and biotechnology. Figure 3 summarizes the development of these biosensors from cell growth to field deployment.

The field has benefited from convergent technological progress in synthetic biology, materials engineering, and device miniaturization, effectively addressing key performance limitations. The ability to function in environments containing compounds toxic to living cells has enabled detection of environmental contaminants at regulatory-relevant concentrations. Simultaneously, advances in preservation techniques have transformed these molecular detection systems into field-deployable tools suitable for use in resource-limited settings.

A notable achievement has been the democratization of advanced analytical capabilities. By eliminating requirements for specialized equipment and trained personnel, these systems help bridge analytical capability gaps between different regions. Integration with widely available electronic devices further enhances their accessibility and deployment potential.

As standardization protocols develop and manufacturing costs decrease, cell-free biosensors are positioned to become key components in global monitoring systems for public health and environmental assessment. Their modular design enables ongoing integration with complementary technologies, with new materials and electronic interfaces expanding their analytical capabilities. The field demonstrates how biochemical engineering can translate fundamental cellular mechanisms into practical analytical tools for detecting diverse analytes under real-world conditions.

## Figures and Tables

**Table 1:** Environmental detection applications.

Target Analyte	Detection Method/System	Limit of Detection	Selectivity/Specificity	Sample Matrix
Mercury [22]	Paper-based, dual- filter, smartphone readout	6 μg/L	Selective for mercury (activation ratio >8–14 for Hg, <2 for others)	Water
Mercury [14]	merR gene, plasmid DNA, firefly luciferase/eGFP	1 ppb	Selective for Hg^2+^; pH optimization and chelating agents enhance specificity	Water
Mercury [24]	Allosteric transcription factors (aTFs)	0.5 nM	High selectivity for target metals; validated in real water samples with 91–123% recovery rates	Water
Lead [24]	aTFs	0.1 nM	High selectivity for target metals; validated in real water samples with 91–123% recovery rates	Water
Lead [25]	Engineered PbrR mutants	50 nM	Selective for lead	Water
Arsenic and mercury [26]	Optimized transcription factors	Arsenic ≤10 μg/L, Mercury ≤6 μg/L	Minimal response to nontoxic ions	Water
Tetracyclines [30]	Riboswitch-based, RNA aptamers	0.4 μM	Broad-spectrum for tetracycline family	Milk samples
Biological warfare agents [32]	16S rRNA targeting, retroreflective particles	1 to 11 fM	Specific for *B. anthracis*, *F. tularensis*, *Y. pestis*, *B. pseudomallei*, *B. abortus*	Laboratory samples
Atrazine [28]	Metabolic pathway and cyanuric acid biosensor	50 μM	Specific for atrazine via metabolic conversion	Laboratory samples

**Table 2:** Medical diagnostic applications.

Target Analyte	Detection Method/System	Limit of Detection	Selectivity/Specificity	Sample Matrix
Thyroid receptor ligands [37]	Allosterically activated fusion protein	48 to 75 nM	β-specific endocrine disruptors	Laboratory samples
Estrogenic compounds [38]	RAPID platform	9 to 330 nM	selective for estrogenic activity	Human blood/urine
3-oxo-C12-HSL [41]	Quorum sensing detection	4.9 nM	Highly specific for 3-oxo-C12-HSL; results comparable to LC-MS	*P. aeruginosa*-infected sputum
Glutamine [7]	Metabolically engineered system	10 μM	Inhibitor-based approach ensures glutamine specificity	Human serum
Bile acids [42]	Transcription factor-based	0.61 μM	Selective for deoxycholic acid	Fecal water, wastewater, serum
Homocysteine [16]	Colorimetric detection	1 μM	Selective detection at clinically relevant thresholds	Plasma
Progesterone [20]	CRISPR-Cas14a + aTFs	67 pM to 0.33 μM	Single-nucleotide discrimination	2 μL sample
Pentachlorophenol [45]	aTF NalC + NASBA	0.002 μM	high specificity for pentachlorophenol; validated with 101–114% recovery	Environmental samples
Protein biomarkers [46]	Split T7 RNA polymerase (TLISA)	50 to 200 nM	Various protein targets	Serum/saliva
SARS-CoV-2 RNA [15]	Paper-based toehold switches	60 nM	High specificity for target RNA	Human saliva

**Table 3:** Complex signal and multiplexed detection systems.

System Type	Detection Method	Limit of Detection	Key Innovation	Sample Compatibility
Signal amplification [60]	Ribozyme cleavage circuits	0.045 μM	Eliminates translation steps	Small molecules
Amplification circuit [76]	Polymerase strand recycling	5 nm to 1 μM	T7 RNA polymerase recycling	Laboratory samples
Electrochemical multiplexing [77]	Gene-circuit-based sensors	65 nM	Electrochemical readouts	Laboratory samples
Multiplexed pathogen detection [78]	Multi-arm RNA junctions	20 aM	Molecular logic operations	Diagnostic samples
Quantum dot multiplexing [79]	Hybrid nucleic acid-QD assemblies	1 enzyme unit ^1^	Multiple enzymatic monitoring	Laboratory samples
CRISPR-based detection [20]	CRISPR-Cas systems integration	4.2 pM	Signal processing enhancement	Complex biological matrices
Pathogen multiplexing [32]	16S rRNA targeting	2.4 nM	Simultaneous multi-pathogen ID	Laboratory samples
Metabolic transducers [33]	Plug-and-play cascades	1 to 10 μM	Synthetic metabolic networks	Complex media/urine

^1^ 1 enzyme unit is described as the amount of enzyme required to cleave 1 μg of DNA at 37 °C.

**Table 4:** Novel sensor design strategies.

System Type	Detection Method	Limit of Detection	Key Innovation	Sample Compatibility
Split reporter system [86]	Split T7 promoter	10 pM	Three-way junction structure	Nucleic acids
Split protein system [87]	Split mNeonGreen	Not reported	Reduced synthesis workload	Laboratory samples
Metabolic biosensing [88]	GABA transaminase + CFPS	47 μM	Enzymatic conversion approach	Laboratory samples
Dopamine detection [84]	Synthetic riboswitches	0.48 μM	Engineered aptamer riboswitches	Human urine
Fluoride detection [85]	Natural riboswitches	0.2 mM	B. cereus crcB riboswitch	Field conditions
Mechanosensitive systems [82]	MscL + biosensing	Not reported	Osmotic pressure integration	Synthetic liposomes
Adaptive synthetic cells [83]	Inducible genetic circuits	Not reported	Mechanosensitivity + gene expression	Synthetic systems
Colorimetric multiplexing [89]	Multi-enzyme systems	Not reported	Visual detection without instruments	Laboratory samples

## Data Availability

Not applicable.

## Abbreviations

The following abbreviations are used in this manuscript:

CFPS	Cell-free protein synthesis
LCMS	Liquid chromatography–mass spectrometry
aTF	Allosteric transcription factor
RAPID	Rapid adaptable portable in vitro detection
TLISA	T7 RNA polymerase-linked immunosensing assay
GABA	γ-aminobutyric acid
SLP	Supported lipid bilayer
